# Development of Intelligent Fault Diagnosis Technique of Rotary Machine Element Bearing: A Machine Learning Approach

**DOI:** 10.3390/s22031073

**Published:** 2022-01-29

**Authors:** Dip Kumar Saha, Md. Emdadul Hoque, Hamed Badihi

**Affiliations:** 1 Department of Mechatronics Engineering, Rajshahi University of Engineering & Technology, Rajshahi 6204, Bangladesh; dip07me@gmail.com; 2 Department of Mechanical Engineering, Rajshahi University of Engineering & Technology, Rajshahi 6204, Bangladesh; mehoque@me.ruet.ac.bd; 3 College of Automation Engineering, Nanjing University of Aeronautics and Astronautics (NUAA), Nanjing 211106, China

**Keywords:** support vector machine (SVM), particle swarm optimization (PSO), fault diagnosis, ball bearing, machine learning (ML)

## Abstract

The bearing is an essential component of a rotating machine. Sudden failure of the bearing may cause an unwanted breakdown of the manufacturing plant. In this paper, an intelligent fault diagnosis technique was developed to diagnose various faults that occur in a deep groove ball bearing. An experimental setup was designed and developed to generate faulty data in various conditions, such as inner race fault, outer race fault, and cage fault, along with the healthy condition. The time waveform of raw vibration data generated from the system was transformed into a frequency spectrum using the fast Fourier transform (FFT) method. These FFT signals were analyzed to detect the defective bearing. Another significant contribution of this paper is the application of a machine learning (ML) algorithm to diagnose bearing faults. The support vector machine (SVM) was used as the primary algorithm. As the efficiency of SVM heavily depends on hyperparameter tuning and optimum feature selection, the particle swarm optimization (PSO) technique was used to improve the model performance. The classification accuracy obtained using SVM with a traditional grid search cross-validation (CV) optimizer was 92%, whereas the improved accuracy using the PSO-based SVM was found to be 93.9%. The developed model was also compared with other traditional ML techniques such as k-nearest neighbor (KNN), decision tree (DT), and linear discriminant analysis (LDA). In every case, the proposed model outperformed the existing algorithms.

## 1. Introduction

Machines are the heart of any industrial unit or manufacturing plant. Numerous types of machinery are available in the industry. The profit of any production plant is highly dependent on the available runtime of machines. A reduction in downtime is essential to increase the company’s profit margin because maintenance costs carry about 15–20% of total production costs [1]. However, it is a reality that almost 30% of maintenance costs are simply wasted due to improper maintenance strategy adoption and failure to perform maintenance at appropriate times. The sudden collapse of machine components may lead to substantial production losses. Proper condition monitoring of these components is essential to ensure the uninterrupted operation of industries. Condition monitoring deals with both present and past aspects of the machines. Various forms of information such as vibration, noise, temperature, current drawn by the motor, and lubricating oil conditions are obtained from the machines during this process. Obviously, this information can play a significant role in developing a suitable maintenance strategy.

Although every machine is essential to keep the plant in operation, only 5–10% of machinery is considered most critical. Compressors, turbines, boilers, generators, and motors are some of the examples of essential components. Statistics show that electrical motors are used as prime movers in more than 90% of the mechanical drives such as gearboxes, compressors, and pumps [2]. The failure in these motors typically falls in the category of bearing-related faults, stator winding associated faults, and rotor-related faults. Among these faults, most of them, almost 40%, occur due to bearing-related issues [3].

Many diagnosis techniques have been developed on the basis of vibration analysis [4], temperature analysis [5], wear debris analysis, motor current signature analysis [6], and acoustic emission analysis [7]. Among these techniques, vibration analysis is considered the most effective, as it can provide significant information about anomalies. A wide-ranging literature investigation on condition monitoring systems (CMSs) thorough vibration analysis was carried out to find possible research gaps. In 2019, Leao et al. developed a fault detection method using the model state observer approach [8]. This method was used for detecting transversal cracks in a horizontal rotating shaft. The authors extracted time-domain features from a raw vibration signal. However, the main problem of this method is that it is not suitable for diagnosing faults when multiple faults occur at the same frequency. Zarei et al. used an intelligent filter for the classification of bearing faults. Three types of faults, inner race fault, outer race fault, and double hole in the outer race, were detected using this technique. The external noise did not have any impact on the developed model performance, which was a vital achievement of this work. having said that, the mandatory requirement of domain knowledge was the drawback of this model [9]. In 2017, Marins and his team developed a classification model based on a similarity-based approach. A publicly available dataset from CWRU (Case Western Reserve University) was used to validate this model. This dataset consists of bearing fault data at different sampling frequencies. In this case, domain knowledge is important [10]. The majorization–minimization-based compound fault diagnosis technique was developed by Hao et al. in 2019 [11]. This method became popular due to its capability of detecting compound faults. However, its runtime was pretty high. A decision tree-based bearing fault classification method was developed by Tahi et al. in [12] to detect misalignment, bearing defects, and unbalancing issues. The genetic wrapper was used in this model for feature selection purposes, where kurtosis and crest factor were considered as the main features. Wind turbine gearbox faults were diagnosed in [13] by Inturi et al., where discrete wavelet transform (DWT) was used as the main feature. A decision tree algorithm was used to build the classification model [13]. However, their classification work was limited to the identification of inner and outer race faults, and other types of faults were not considered in this work. Features are the main resource of the machine learning model. However, excessive features sometimes create complexity in model development. On that note, feature reduction can play a vital role in improving the accuracy of classification. Zhao et al. developed a model through a feature reduction technique with global–local margin Fisher analysis [14]. Here, a Euclidean weighted K-nearest neighbor algorithm was mainly used as a classification algorithm. In 2015, Gowid et al. [15] developed a novel condition monitoring technique using acoustic emission. This model was applied to the industrial blower dataset to identify the fault. In this model, spectral features were extracted from raw acoustic emission signals. Rauber et al. [16] used envelop spectrum with statistical time- and frequency-domain features to diagnose the fault in bearing. However, this approach produces a large number of heterogeneous features and, at the same time, creates irrelevant and redundant information that generates complexity in the model and ultimately increases the computational cost [16]. Frequency spectrum [17] and pattern recognition [18] are the two most critical analyzing features for identifying bearing faults. However, the superiority of pattern recognition over the spectrum approach was shown in [19]. In machine learning model development, feature selection is considered as one of the most critical factors. Sometimes, irrelevant features create complexity in model development. To extract the optimum feature subset from raw datasets, the minimum redundancy maximum relevance (nRMR) method was developed in [20]. Due to its effectiveness in real-time analysis and excellent generalization performance, the support vector machine (SVM) was successfully applied by He et al. for signal processing purposes [21] and by Chen et al. for pattern recognition [22]. In SVM, kernel function selection is an important task [23]. Various types of kernels are available to transform the low-dimensional space into a high-dimensional space. Having said that, due to its capability in approximating nonlinear functions, the radial basis function (RBF) is considered the most effective. During model development, another important issue is the so-called hyperparameter tuning. The most convenient approach that researchers usually follow is the trial-and-error method. The main drawback, however, is that it involves a manual iteration process that ultimately increases the computational time. Another approach often followed by researchers is using a grid search CV; however, it suffers from a low operating speed and lower accuracy. This is due to the handling of a large number of parameter combinations. Another technique is using evolutionary algorithms that belong to the metaheuristic family [24]. Huang et al. [25] proposed an optimization method based on genetic algorithm (GA) to optimize the SVM parameter and feature selection process. Lee et al. [26] developed a bearing fault diagnosis method based on ensemble empirical mode decomposition and principle component analysis. In this paper, a particle swarm optimization technique was used to improve the SVM model’s computational efficiency, which is one of the main goals of this research. The PSO has a quick convergence capability, parallel computation ability, and very fey parameters to handle [27]. We are now passing into the era of big data and the Internet of things (IoT). To handle these huge data is not easy, especially for industrial applications, where there is a wide variation in data characteristics. Data fusion is one of the prominent processes that can minimize the problem to some extent [28]. Luwei et al. developed an integrated fault detection framework using the data fusion process of frequency-domain data [29]. In 2020, Wei et al. developed a fault diagnosis of a complex system using the data fusion technique [30]. In this paper, the faults were identified at the earliest time when the defect occurred. In the same year, a composite spectrum-based multisensor data fusion technique was adopted by Akilu and Ruifeng to detect faults on rotating machinery [31]. Banerjee et al. developed a motor fault detection technique using SVM as the primary algorithm with a multisensory data fusion technique [32]. Although it was published in 2012, its contribution is still considered a remarkable one. Cao et al. developed a gear fault detection model with data fusion in 2021 [33]. Carlos et al. proposed a vibration-based fault diagnosis technique for induction motors using the orthogonal matching pursuit algorithm [34].

One main challenge in developing an effective condition monitoring technique is the selection of a proper approach. The maximum model-based approach provides lower accuracy when the machine is complex, whereas the data-driven machine learning approach is promising. A suitable dataset is the main requirement of building a classification algorithm. There are a few popular datasets such as the “NASA repository” and the “PROGNOSTIA” experimental platform. However, in this research, a fresh dataset was used. This setup created opportunities to extract data in various operating conditions. This unit system consisted of a three-phase induction motor with variable-speed operating capability, rotary machine elements bearing, and a power transmission system. One of the system’s major advantages is its very low or no self-vibration feature. The bearing was mounted in the system in such a way that it could be easily replaced on a plug-and-play basis. There was also scope to change the balancing mass of the system. Although only bearing fault diagnosis was considered in this work, it is possible to generate gear pitting fault data using this system. This paper also uses some time-domain features which were not investigated previously.

The main contributions of the paper can be outlined as follows:

- An experimental setup is designed and developed for real-time data generation purposes based on which the proposed ML model is developed.
- A novel hybrid PSO–SVM model is proposed for improving the generalization capability of the SVM algorithm through optimization of hyperparameters (C and γ) of the radial basis function (rbf).
- The PSO optimization technique is used to improve the classification accuracy of different bearing faults.
- A comparative analysis is shown with popular machine learning algorithms to validate the proposed model. Results show a significant improvement in model performance due to the introduction of PSO.

## 2. Experimental Setup

**Figure 1:**
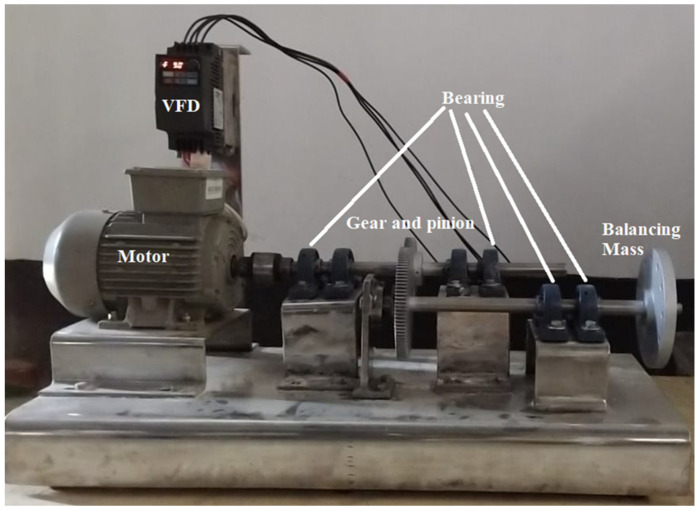
Photograph of the experimental setup.

The development of the experimental setup was one of the major challenges of this research work. It was essential to ensure the reliability of various components used for the development of this setup. It has always been maintained to use quality components because a quality system can only produce quality data. The efficiency of the machine learning approach is heavily dependent on data quality. A photograph of the real experimental setup is shown in Figure 1. A three-phase induction motor with 0.5 Hp rated power and up to 6000 RPM rotating speed was used as the driving source of the experimental design. A variable frequency drive (VFD) was installed with a rated frequency of 0 to 599 Hz, just before the induction motor, to run the machine in different operating conditions. The power supply from the primary power source was provided to the motor through this VFD. The shaft was connected with the motor through a coupling. Four bearings carried both the static and dynamic load of the shaft. The bottom structure was constructed with stainless steel to increase the load-carrying capacity and avoid rust problems. Flexible coupling and mounting systems were adopted for both the bearing and shaft to address easy replacement purposes. Although it was possible to create faults in all six bearings, only one bearing was considered to maintain a benchmark and avoid complexity in data generation. A vibration analyzer was used with a measuring range of ±5 V AC, a frequency range of 2 to 10 kHz, and a maximum sampling frequency of 25.6 kHz. The acceleration data were measured with the help of a Ronds accelerometer sensor with a measuring range of ±80 g, frequency range of 0.7 to 10,000 Hz, and resonance frequency of about 30 kHz.

## 3. Methodology

**Figure 2:**
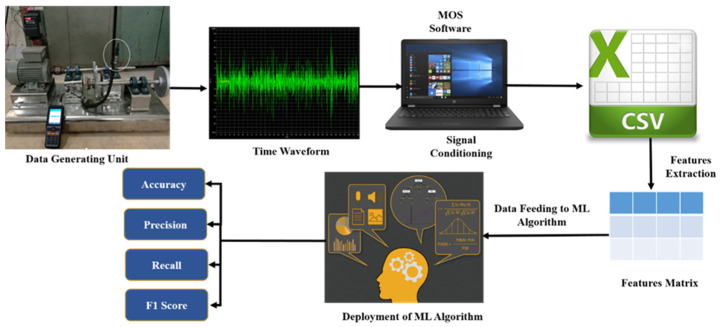
Step-by-step approach from data generation to fault diagnosis.

The overall methodology of the paper can be segregated in several stages as shown in Figure 2. All stages are explained below.

**Figure 3:**
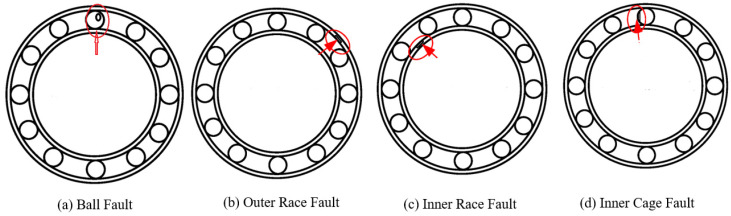
Typical bearing faults.

(a) Data generation: This is the most essential part of this methodology section. As already mentioned, in order to avoid complexity in data generation, only one bearing was considered as the testing bearing. The common bearing faults that occurred in deep groove ball bearing are “ball fault” (BF), “outer race fault” (OR), “inner race fault” (IF), and “cage fault” (CF), as shown in Figure 3. All the healthy and faulty bearing system data were collected according to the predefined data generation plan, as shown in Table 1. Here, Outer_Race_10 (OR-10) denotes the dataset of outer race faults collected at 10 Hz rotational frequency. The generated vibration data were collected using an accelerometer sensor mounted to the bearing housing. All faults were seeded manually in our workshop.
(b) Signal conditioning: It is not always possible to have experimental data in the desired format. Usually, the data accusation system maintains the data in a time waveform. This time waveform signal is then converted into “.txt” and ultimately into “.csv” formats using MOS 3000 software (provided by Anhui Ronds Science and Technology Inc., Anhui, China).
(c) Feature extraction: This is one of the most critical steps when developing any ML model, as the model’s performance primarily depends on the selection of features. Various time-domain features such as kurtosis, crest factor, and form factors, are extracted from raw time-domain signals using their corresponding governing equations [35]. A feature matrix was prepared with extracted time-domain features.
(d) Model development: This is the core part of this methodology section. A section dedicated to the machine learning approach for fault detection is introduced in Section 4 to give a detailed idea about the mathematical model.
(e) Model prediction: Lastly, the model was used to predict the classification results in terms of accuracy, precision, recall value, and F1 score.

## 4. Machine Learning Approach for Fault Detection

### 4.1. Support Vector Machine (SVM)

In this paper, a multiclass support vector machine (SVM) was used as the classification algorithm. SVM works on the principle of structural risk management (SRM) [36]. It is best suited for classification tasks when the sample size is not too big. The primary concept is to find the optimal hyperplane that differentiates the training data into two binary classes by maximizing the margin. In a multiclass classification problem, the datasets other than the target one are combined to create a binary classification situation. The data points located near imaginary lines are known as support vectors. For any given sets of data (*u*_1_, *v*_1_), (*u*_2_, *v*_2_),…, (*u_n_*, *v_n_*), $u_{i}\in \;R^{n}$ are considered as inputs, where u and v represent respective data points of support vectors, and $v_{i}\in \left(-1,+1\right)\;$are the outputs for each *u_i_*. The equation of the hyperplane can be represented as follows:

(1) (w·u)+b=0,

where *w* is a vector with dimension N, and *b* is a scalar quantity. In more generalized forms, the equations of the hyperplane for *v_i_* = 1 and *v_i_* = −1 can be represented, respectively, as follows:

(2) $$\left(w\cdot u_{i}\right)+b\geq 1,$$

(3) $$\left(w\cdot u_{i}\right)+b\leq 1.$$

For any positive margin,

(4) $$\left(w^{T}\cdot u_{2}\right)+b=+1.$$

For any negative margin,

(5) $$\left(w^{T}\cdot u_{1}\right)+b=-1.$$

By subtracting Equation (5) from Equation (4), one gets the maximum margin as follows:

(6) $$w^{T}\left(u_{2}-u_{1}\right)=2,$$

(7) $$\frac{w^{T}}{\left\|w\right\|}\cdot \left(u_{2}-u_{1}\right)=\frac{2}{\left\|w\right\|}.$$

This is the optimized function that needs to be maximized.

Error optimization: For any new testing data *v_i_*, one gets

(8) $$v_{i}*\left(w^{T}\cdot \;u_{i}\right)+b_{i}\geq 1.$$

If the above condition is not satisfied, it indicates a case of misclassification. In that case, it is necessary to include the error term to calculate *w* and *b*.

(9) $$(w^{*},\;b^{*})=\min\frac{\left\|w\right\|}{2}+C_{i}\overset{n}{\underset{i=1}{\sum }}\varepsilon_{i},$$

where *C* is the error penalty, and ε is the slack variable.

### 4.2. Particle Swarm Optimization (PSO)

The concept of particle swarm optimization (PSO) came from analyzing the social behavior of animal groups. Research has shown that some animals such as birds and fishes can share information with their group members while traveling. This finding inspired Kennedy and Eberhart to develop PSO, a metaheuristic algorithm that can optimize nonlinear continuous functions [37]. The idea of swarm intelligence is typically observed in flocks and shoals, i.e., groups of animals.

PSO aims to determine a variable denoted by X=[x1, x2, x3, ……xn] that optimizes the parameters on the basis of function f(x). Here, f(x) is a fitness function or objective function; *X* represents an *n*-dimensional position vector, where *n* is the number of variables. The position of each particle is assessed on the basis of the value of the fitness function. The PSO is conducted using the following equations:

The particle velocity is updated by

(10) $$v_{ij}^{*}\left(t+1\right)=w^{*}v_{ij}^{*}\left(t\right)+c_{1}r_{1}\left(pbst_{ij}-x_{ij}^{*}\left(t\right)\right)+c_{2}r_{2}\left(gbst_{ij}\left(t\right)-x_{ij}^{*}\left(t\right)\right).$$

The particle position is updated by

(11) $$x_{ij}^{*}\left(t+1\right)=x_{ij}^{*}\left(t\right)+v_{ij}^{*}\left(t+1\right).$$

The fitness function, which indicates the average classification of the model, is calculated by

(12) $$f\left(x\right)=\frac{1}{n}\overset{n=n}{\underset{n=1}{\sum }}\frac{\mathrm{TP}+\mathrm{TN}}{\mathrm{TP}+\mathrm{TN}+\mathrm{FP}+\mathrm{FN}}\;,$$

where $v_{ij}^{*}\left(t\right)$ is the *i*-th particle’s velocity at the *t*-th iteration, $pbst_{ij}\left(t\right)$ indicates the best position of the particle, and $gbst_{ij}\left(t\right)$ indicates the particle’s best position among all. TP means “true positive”, TN means “true Negative”, FP means “false positive”, and FN means “false negative”.

### 4.3. PSO–SVM Model Construction

In this paper, a particle swarm optimizer was used for SVM hyperparameter optimization purposes. The initialization of the PSO parameters is important for faster optimization, and it was conducted as outlined in Table 2.

The step-by-step process of the PSO–SVM model is described as follows:

Step 1: Preprocess the raw vibration data to have them in usable form.

Step 2: Extract time-domain features and split the data into training and testing datasets. Perform cross-validation to generate multiple feature subsets.

Step 3: Initialize the PSO parameters as in Table 2.

Step 4: Generate the initial position and velocity of the swarm particle using Equations (10) and (11).

Step 5: For the first iteration *t* = 1, calculate the fitness function using Equation (12) with a 10-fold cross-validated training dataset. A high fitness value denotes low error in classification problems.

Step 6: Update the velocity and position of each particle. Terminate the process when *t* = *t*max. Select the best value of γ and C. Train the model on the basis of this value.

Step 7: Test the model with test datasets. Find the test accuracy of the model along with the classification report.

**Figure 4:**
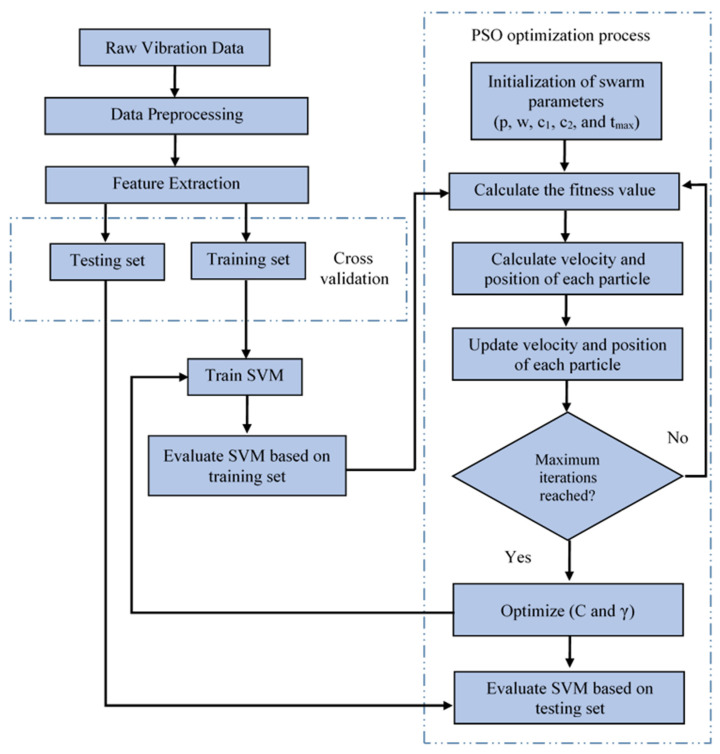
Flow diagram of PSO–SVM algorithm.

The flowchart of the PSO–SVM model is shown in Figure 4.

### 4.4. Confusion Matrix

**Figure 5:**
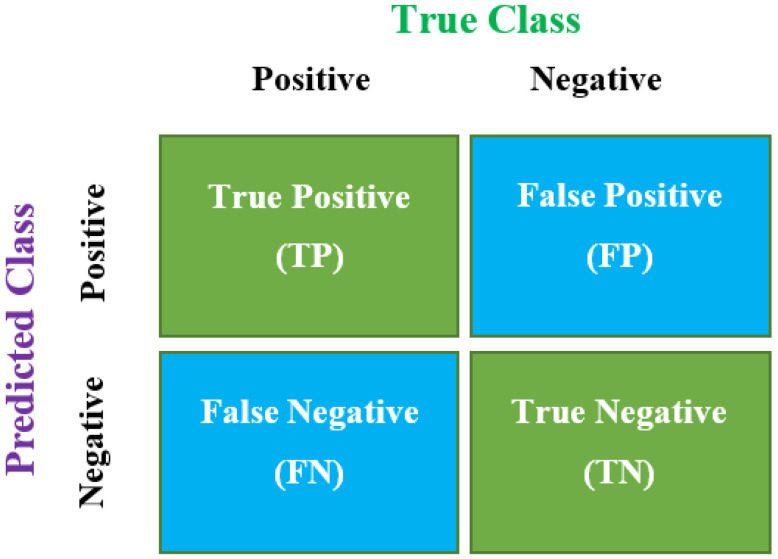
Confusion matrix.

A confusion matrix is a table capable of describing the performance of a machine learning model for classification purposes for a particular set of data where true values are known. It can be used for both binary and multiclass classification purposes, as shown in Figure 5.

#### 4.4.1. Accuracy

Accuracy is one of the essential performance parameters for classification algorithms. It can be defined as the ratio of the number of correctly predicted data to all predictions made. It may be calculated using the following formula:

(13) $$\mathrm{Accuracy}=\frac{\mathrm{TP}+\mathrm{TN}}{\mathrm{TP}+\mathrm{TN}+\mathrm{FP}+\mathrm{FN}}.$$

#### 4.4.2. Precision

Precision may be defined as the ratio of actually predicted true values to the total number of values predicted as true.

(14) $$\mathrm{Precision}=\frac{\mathrm{TP}}{\mathrm{TP}+\mathrm{FP}}.$$

#### 4.4.3. Recall

Recall may be defined as the ratio of true positive values to the sum of true positive and false negative values. It calculates how many of the actual positives the model captures by labeling them as true positives. It can be calculated using the following formula:

(15) $$\mathrm{Recall}=\frac{\mathrm{TP}}{\mathrm{TP}+\mathrm{FN}}.$$

#### 4.4.4. F1 Score

When a comparison is made between two models where they have high recall value but low precision or vice versa, it becomes difficult to evaluate which one is best. In this case, a third parameter, namely, the F1 score, makes them comparable.

(16) $$F1\;\mathrm{score}=2*\;\frac{\mathrm{Precision}*\mathrm{Recall}}{\mathrm{Precision}+\mathrm{Recall}}.$$

## 5. Results and Discussion

### 5.1. Experimental Data

**Figure 6:**
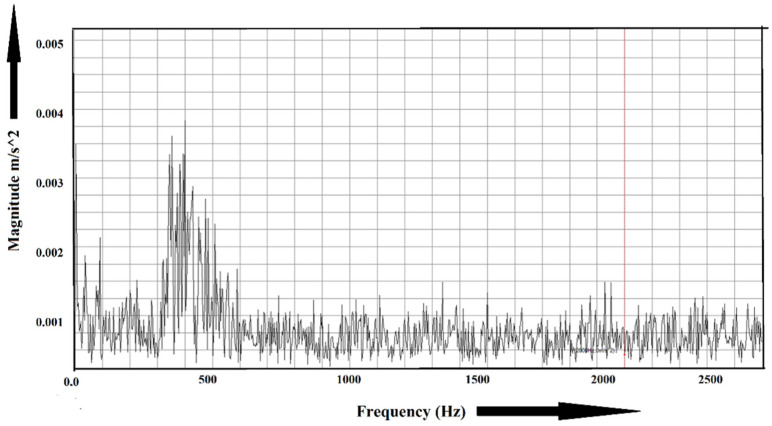
Frequency spectrum of a healthy bearing.

**Figure 7:**
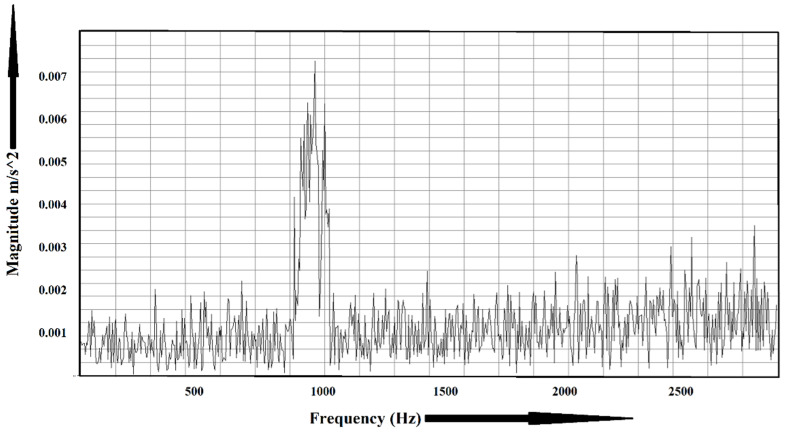
Frequency spectrum of a faulty bearing.

Some sample experimental data are shown in Figure 6 and Figure 7. Figure 6 shows the frequency spectrum of a healthy bearing, and the faulty condition is shown in Figure 7. For the healthy bearing, the maximum amplitude was found to be $0.0035\;m/s^{2}$, whereas, for the faulty bearing, the amplitude went up to $0.006\;m/s^{2}$. Fast Fourier transform (FFT) plays a vital role in identifying faults in machinery. However, it loses its efficiency when multiple faults occur at the same frequency.

### 5.2. Data Preprocessing

**Figure 8:**
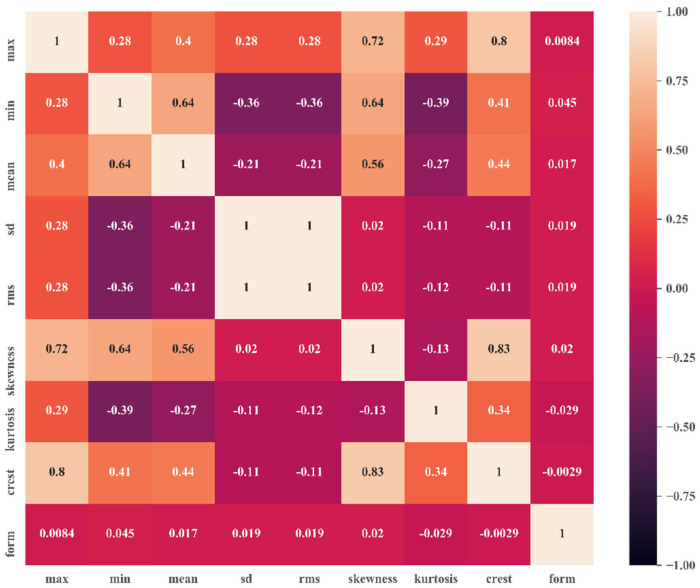
Correlation matrix of extracted time−domain features.

Data preprocessing involves filtering, handling missing values, feature extraction, signal processing, data normalization, data scaling, and selecting the optimum feature subset. Figure 8 illustrates the correlation matrix of various features extracted from raw time-domain signals. Here, it can be observed that the RMS and standard deviation were closely related to each other. The crest factor and skewness also showed a good correlation. The mean and RMS offered very low correlation, with close to no relation. The optimum parameters were selected on the basis of this correlation matrix, which was essential to create optimum decision boundaries.

### 5.3. Model Performance

**Figure 9:**
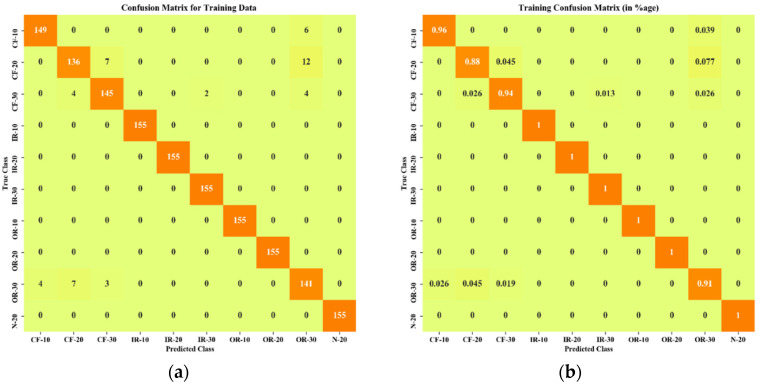
Confusion matrix of training data: (**a**) in terms of number of training data points; (**b**) in terms of percentage (%).

**Figure 10:**
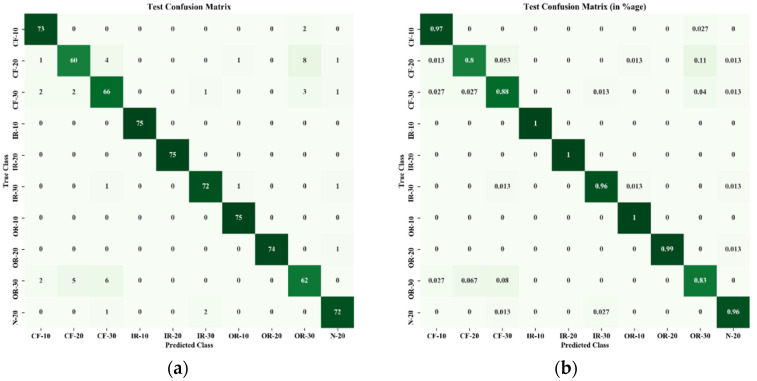
Confusion matrix of testing data: (**a**) in terms of number of testing data points; (**b**) in terms of percentage (%).

Figure 9 and Figure 10 show the confusion matrices obtained from the proposed method for training and testing data. A description of data labels and supportive information is given in Table 1. In total, 230 feature data were segmented into two sets, 155 data points for the training set and 75 data points for the testing set. The horizontal axes were assigned as predicted classes, and vertical axes were used as true classes. Figure 9a shows the confusion matrix in terms of sample data of the training set, and Figure 9b expresses the classification results in terms of percentage. It is observed that, out of 10 classes, six classes of faults (IR-10, IR-20, IR-30, OR-10, OR-20, N-20) were identified perfectly without any misclassifications. In the case of cage faults (CF-10 and CF-30), very few misclassifications were observed. Figure 9 shows the confusion matrix of the testing set. Training accuracy also showed promising characteristics by classifying three faults perfectly (IR-10, IR-20, and OR-10) without any misclassification, as shown in Figure 10a. From Figure 10b, it can be noted the maximum number of misclassifications (total 15) was found for CF-20.

The classification report is shown in Table 3. Such a classification report can be used to assess the accuracy of a classification algorithm’s prediction performance. The precision value indicates the ratio of the actual predicted true value to the total predicted true value. When the value is close to 1.0, the model is described as a highly precise model, whereas, with low precision, its value becomes close to 0.0. The proposed model showed higher precision for inner race faults with both 10 and 20 rotational speeds. Furthermore, the outer race fault with a 10 Hz frequency was precisely identified. Similar data were also obtained for recall value.

A performance comparison of the proposed model with existing models applied to the bearing datasets is shown in Table 4. It can be observed from the results that the SVM with grid search CV produced 92% testing accuracy, whereas an almost 2% improvement in accuracy was obtained using SVM with PSO, which is far better than other models. The decision tree and KNN attained good accuracy; however, their range of accuracy was 84–85%. The linear discriminant analysis (LDA) result was not satisfactory, as its accuracy was only 73.7%. Due to considerable robustness to noisy training data and less vulnerability to overfitting issues, SVM was superior to other ML algorithms.

## 6. Conclusions

In this paper, a data-driven intelligent fault diagnosis technique was developed for early fault detection of the deep groove ball bearing. An experimental setup was fabricated considering all the design criteria to produce data similar to real-life industry data. The experimental data were analyzed to separate the faulty bearing from the healthy one as a function of the fault magnitude. During experimentation, data were collected in a silent condition to avoid external noise, and the base was properly mounted to avoid any self-vibrations. The time wave data were captured in image format. These image data were converted into numeric data using the default computational tool of the vibration analyzer. The raw data were then passed through various signal conditioning processes, i.e., data filtering, missing data handling, data scaling, etc. Time-domain features were then extracted, and optimum features were selected on the basis of the relations in the correlation matrix. The correlation matrix showed that the RMS and standard deviation had a greater correlation than other pairs with an absolute value of 1. The relation between crest factor and skewness also looked promising with a correlation value of 0.83. The feature matrix was then fed into the PSO–SVM model. The classification accuracy obtained by PSO-based SVM was 93.9%, almost 2% greater than the accuracy obtained by SVM using the traditional grid search CV method. This improved performance of the proposed hybridized method was achieved due to the extreme robustness to noisy training data and less vulnerability to overfitting issues of the support vector machine algorithm. To validate the efficiency of the PSO–SVM algorithm, the model’s performance was compared with KNN, DT, and LDA using the same bearing dataset. The comparative analysis shows that the proposed model outperformed all other traditional algorithms by a large margin, with almost 10–20% more accuracy. Although this work obtained significant accuracy in bearing fault classification, there is still an opportunity to improve its performance. The application of deep learning or transfer learning methods represents an option. Due to manufacturing difficulty, the ball fault was not considered in this research; thus, it may be included in future work. The experimental setup developed for data generation of bearing faults can also be used for gear pitting fault data preparation purposes. Therefore, there is also scope to work on gear fault classification in future research.

## Figures and Tables

**Table 1:** Data generation plan.

SN	Bearing Conditions	Parameter	Motor Speed (Hz)
01	Normal condition	Acceleration	20
02	Outer-race fault	Acceleration	10, 20, 30
03	Inner-race fault	Acceleration	10, 20, 30
04	Cage fault	Acceleration	10, 20, 30

**Table 2:** Initialization parameters of PSO–SVM model.

SN	Bearing Conditions	Symbol	Initial Value
01	Population size	p	25
02	Inertia weight	w*	1
03	Acceleration coefficient 1	*c*_1_	1
04	Acceleration coefficient 2	*c*_2_	2
05	Maximum iterations	*t*max	150
06	Cross-validation number	k	10
07	Search range of error penalty	*C*	0.1 to 100
08	Search range of kernel parameter	γ	0.001 to 6

**Table 3:** Classification report of PSO-SVM classifier.

SN	Fault Types	Precision	Recall	F1-Score
01	Cage_Fault_10	0.9358974359	0.9733333333	0.9542483660
02	Cage_Fault_20	0.8955223881	0.8000000000	0.8450704225
03	Cage_Fault_30	0.8461538462	0.8800000000	0.8627450980
04	Inner_Race_10	1.0000000000	1.0000000000	1.0000000000
05	Inner_Race_20	1.0000000000	1.0000000000	1.0000000000
06	Inner_Race_30	0.9600000000	0.9600000000	0.9600000000
07	Normal_20	0.9740259740	1.0000000000	0.9868421053
08	Outer_Race_10	1.0000000000	0.9866666667	0.9932885906
09	Outer_Race_20	0.8266666667	0.8266666667	0.8266666667
10	Outer_Race_30	0.9473684211	0.9600000000	0.9536423841
	Accuracy	0.9386666667		
	Macro average	0.9385634732	0.9386666667	0.9382503633
	Weighted average	0.9385634732	0.9386666667	0.9382503633

**Table 4:** Comparative analysis of the proposed model with other algorithms.

SN	Name of Machine Learning Model	Classification Accuracy (Testing) in %
01	K-nearest neighbor (KNN)	84.3
02	Decision tree (DT)	85.3
03	Linear discriminant analysis (LDA)	73.7
04	SVM with grid search CV	92
05	SVM with PSO (proposed model)	93.9

## Data Availability

All data are available in the paper. However, any additional data are available to the readers upon reasonable request to the corresponding authors.
